# Progression of Regional Microstructural Degeneration in Parkinson’s Disease: A Multicenter Diffusion Tensor Imaging Study

**DOI:** 10.1371/journal.pone.0165540

**Published:** 2016-10-31

**Authors:** Yu Zhang, I-Wei Wu, Duygu Tosun, Eric Foster, Norbert Schuff, the Parkinson’s Progression Markers Initiative

**Affiliations:** 1 Department of Veteran Affairs Medical Center, San Francisco, California, United States of America; 2 Department of Radiology and Biomedical Imaging, University of California, San Francisco, California, United States of America; 3 Department of Biostatistics, College of Public Health, University of Iowa, Iowa City, Iowa, United States of America; University of Manchester, UNITED KINGDOM

## Abstract

This study aimed to identify the utility of diffusion tensor imaging (DTI) in measuring the regional distribution of abnormal microstructural progression in patients with Parkinson’s disease who were enrolled in the Parkinson's progression marker initiative (PPMI). One hundred and twenty two de-novo PD patients (age = 60.5±9) and 50 healthy controls (age = 60.6±11) had DTI scans at baseline and 12.6±1 months later. Automated image processing included an intra-subject registration of all time points and an inter-subjects registration to a brain atlas. Annualized rates of DTI variations including fractional anisotropy (FA), radial (rD) and axial (aD) diffusivity were estimated in a total of 118 white matter and subcortical regions of interest. A mixed effects model framework was used to determine the degree to which DTI changes differed in PD relative to changes in healthy subjects. Significant DTI changes were also tested for correlations with changes in clinical measures, dopaminergic imaging and CSF biomarkers in PD patients. Compared to normal aging, PD was associated with higher rates of FA reduction, rD and aD increases predominantly in the substantia nigra, midbrain and thalamus. The highest rates of FA reduction involved the substantia nigra (3.6±1.4%/year from baseline, whereas the highest rates of increased diffusivity involved the thalamus (rD: 8.0±2.9%/year, aD: 4.0±1.5%/year). In PD patients, high DTI changes in the substantia nigra correlated with increasing dopaminergic deficits as well as with declining α-synuclein and total tau protein concentrations in cerebrospinal fluid. Increased DTI rates in the thalamus correlated with progressive decline in global cognition in PD. The results suggest that higher rates of regional microstructural degeneration are potential markers of PD progression.

## Introduction

Parkinson’s disease (PD) is a slowly progressing neurodegenerative movement disorder clinically characterized by rigidity, tremor, and bradykinesia that affects about ten million people worldwide [1]. While there is currently no cure for PD, various neuroprotective agents are being developed to slow or halt disease progression and enhance life quality of patients. However, the discovery of an accurate biomarker of disease progression, which is critical in the search for disease modifying interventions, has been elusive. The Parkinson’s Disease Progression Marker Initiative (PPMI) is an observational clinical study that includes brain imaging to validate potential biomarkers of PD [2]. We investigated whether PD patients enrolled in the PPMI exhibit a progressive decline in microstructural integrity—based on magnetic resonance diffusion tensor imaging (DTI)—that could offer a surrogate biomarker of disease progression.

Evidence for an involvement of microstructure in PD comes from neuropathological studies that indicate Lewy neurites and Lewy bodies, hallmarks of PD, evolve according to a predictable topographical sequence along major fiber pathways, beginning in the brain stem and eventually advancing to neocortical regions [3]. Several cross-sectional DTI studies in PD also implied microstructural abnormalities in various brain regions (for review see [4]). In contrast, longitudinal diffusion MRI studies in PD have been rare [5–8]. Two longitudinal studies have described diffusion changes in the substantia nigra of PD. First, Ofori et al. [7] used a bi-tensor model and found that free-water diffusion increased in the substantia nigra in PD patients over a one year period, whereas the control group showed no significant change. The second study by Loane et al. [8] followed PD patients over a period of 19.3 months and found that a single tensor model identified decreasing fractional anisotropy within the substantia nigra over time. The current study aims to extend this prior work by examining other parts of the brain in a dataset that involves a larger sample collected across multiple MRI centers. Furthermore, no study before investigated associations between microstructural changes in PD and other potential biomarkers of PD progression, such as variations in dopamine deficits [9] or specific proteins cerebrospinal fluid (CSF) [10].

We report longitudinal DTI findings from a large cohort of PD patients (n = 122) relative to findings in healthy aging (n = 50). Our specific aims were: (1) to determine the degree and regional distribution of microstructural integrity decline in PD relative to healthy aging; (2) to test whether this PD-specific microstructural decline correlates with the decline of motor and cognitive functions, as well as the progression of PD biomarkers, such as changes in dopamine deficit and in CSF α-synuclein concentration.

## Materials and Methods

### Subjects

**Fig 1:**
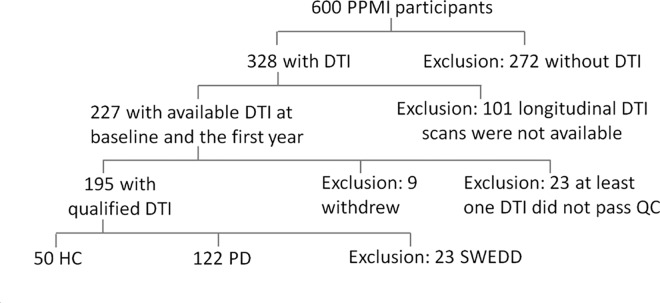
Sample selection process.

PD patients and healthy control (HC) subjects in this study were enrolled in PPMI. In brief, PPMI is an ongoing observational, international, multicenter study (16 US sites, 5 European, 1 Australian) aimed to identify serological, genetic, spinal fluid and imaging biomarkers of PD progression in a large cohort of newly diagnosed PD patients. The study was launched in June 2010 and has successfully completed its enrollment goal of 400 PD patients and 200 HC subjects. At baseline, PD patients had to be older than age 30, diagnosed with PD within the past 2 years, have a Hoehn and Yahr (H&Y) [11] stage not larger than II, be untreated, and have shown dopamine deficiency on ^123^Ioflupane dopamine transporter (DAT) imaging [12]. Of those subjects 227 underwent a standardized DTI protocol (www.ppmi-info.org) at baseline and after 12 months. The selection of these subjects was driven by the technical capabilities of the PPMI participating centers to obtain DTI in a rigorous and standardized fashion. Subjects were excluded if they withdrew from the study, failed image quality, or were diagnosed with PD but had no evidence of dopaminergic deficit on DAT, the so-called SWEDD group. Finally, 172 subjects (PD = 122, HC = 50) who had two sequential MRI scans on average 12.6 ± 1 months apart were included. A graphical summary of the subject selection is shown in Fig 1. This study has been approved by the respective institutional review boards of all participating sites including Baylor College of Medicine, The Parkinson's Institute, John Hopkins University, Emory University, Northwestern University Medical School, Mellen Center Cleavland Clinic, Boca Raton Regional Hospital, University of Tubingen, Paracelsus-Elena Klinik, University of Innsbruck, and all subjects provided written informed consent.

### Clinical assessments and dopaminergic imaging

The subjects were assessed with a wide spectrum of clinical tests at every study visit, including the Movement Disorders Society Unified Parkinson's Disease Rating Scale (MDS-UPDRS) [13] and the Montreal Clinic Assessment (MoCA) for mild cognitive impairment [14]. For patients who had started dopaminergic therapy during the follow-up period, the tests are assessed in both the defined OFF state (12 hours post last dose of medication) and the ON state (approximately an hour after the last dose of medication). All subjects also had DAT imaging at baseline and PD patients had DAT imaging again after 12 months, as routinely acquired in the striatum using ^123^Ioflupane single-photon emission computed tomography (SPECT). The DAT images were centrally reconstructed, attenuation corrected and analyzed with a standardized volume of interest template for extraction of regional count densities (http://www.indd.org/). Putaminal DAT binding ratios were calculated from SPECT imaging as DAT counts in the left and right putamen DAT counts from the occipital lobe as reference (http://www.indd.org/).

### Cerebrospinal fluid biomarker assessments

Cerebrospinal fluid (CSF) collection was performed at each study site as described in the PPMI biologics manual (http://www.ppmi-info.org/). CSF β-amyloid (Aβ_1–42_), tau proteins total (t-Tau) and phosphorylated (p-Tau_181_) concentrations were analyzed at the University of Pennsylvania as previously described [15]. CSF α-synuclein concentration was analyzed at Covance (Princeton NJ) using a commercially available enzyme-linked immunosorbent assay kit [16]. For quality reasons, the CSF collections were also analyzed for blood contamination and to control for the possible effect of hemolysis on the CSF protein level [17].

### MRI processing

**Fig 2:**
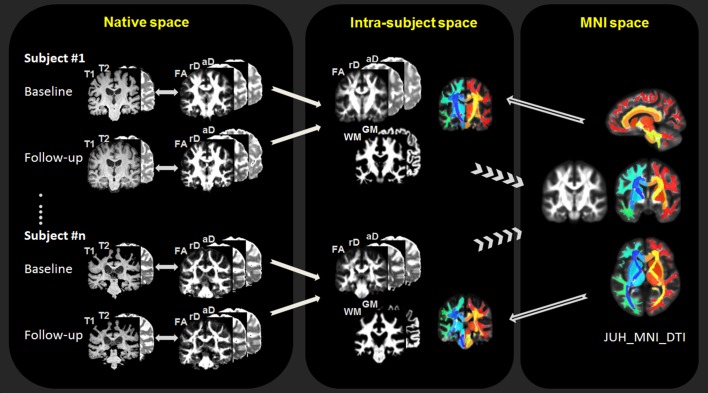
Flowchart of longitudinal DTI data processing. FA = fractional anisotropy; rD = radial diffusivity; aD = axial diffusivity; WM = white matter; GW = gray matter. MNI = Montreal Neurological Institute.

For quantitative MRI measurements, the anatomical and DTI images were first visually inspected for egregious image artifacts and then processed using an automated processing script designed for longitudinal data analysis. The workflow is illustrated in Fig 2: 1) The initial steps include corrections for head motion, eddy-current effects and susceptibility distortions of DTI [18], followed by the computation of standard scalar parameter maps of the diffusion tensor, such as fractional anisotropy (FA), radial diffusivity (rD), and axial diffusivity (aD). 2) Next, an intra-subject affine registration is performed between the parametric DTI maps and the structural T1- and T2- weighted images for each time point. To reduce measurement bias toward the chronological order of image acquisitions, the baseline and follow-up images are further registered to a time-averaged template, which is created by performing an initial affine registration between baseline and follow-up images, followed by a fast diffeomorphic registration (DARTEL) in SPM8 [19]. 3) Next, an inter-subject registration is performed for group analysis using the standard protocol of DARTEL, which involves tissue segmentation of the structural images for DARTEL initialization, a diffeomorphic algorithm for inter-subject image registration, and finally a spatial normalization of the registered images to MNI space [20], allowing the anatomical parcellation of the brain according to the JHU-DTI-MNI (Type I WMPM) atlas (http://cmrm.med.jhmi.edu) [21]. To reduce any group bias in the anatomical parcellation, a group-averaged template is created from all subject images in MNI space, followed by a non-linear registration between the JHU-DTI-MNI atlas and the group-averaged template. 4) In the final step, the JHU-DTI-MNI atlas is reversely transformed to each subject space, facilitating regions-of-interest (ROIs) extraction from each parametric DTI map at baseline and follow-up. For group analysis, DTI measures and white matter density (based on tissue-segmentation) were extracted from 118 ROIs in the entire white matter and subcortical regions, including the basal ganglia and brain stem sub-regions. Potential artifacts due to partial volume were reduced by extracting ROIs in DTI conditioned on brain tissue content derived from the corresponding segmented structural MRI data. Specifically, to reduce artifacts due to brain atrophy, the ROIs were extracted from regions with more than 90% probability of brain tissue content. To further reduce artifacts to partial brain tissue volume, a threshold of more than 50% probability of either white matter or gray matter content was applied for ROIs in respectively white matter or gray matter areas.

### Statistical analysis

Statistical tests were carried out using R Project (http://www.r-project.org/). Linear mixed-effects models were used to estimate rates of DTI, clinical, DAT and CSF biomarker concentration changes by group. Each measurement (e.g. clinical measure or regional DTI variation) of all subjects at all time points were entered as the response variable, time variation and interacted by diagnostic group were entered as fixed effects in the model. Variations across subjects were entered as random effects in the form of a random intercept. Age, gender and medication status were added as covariates. Medication status dichotomized patients into a group that chose to take PD medication during the first year of the study and another group that stayed off PD medication, though all patients were free of PD medication at the time of their MRI baseline scans. Because most PD patients in this study (95%) had unilateral onsets of motor symptoms, a side-by-time interaction term was included in linear mixed-effects models to further test laterality of DTI changes. Side was defined as ipsilateral or contralateral region, according to each patient’s clinically dominant side of Parkinsonian symptoms onset. For all HC subjects and one PD patient with symmetrical symptom onset, left and right DTI values were averaged [18]. Associations between regional DTI changes and changes in clinical scores, DAT imaging, or CSF α-synuclein concentrations in PD patients were assessed using Pearson’s product moment correlation tests. To limit the number of possible tests, these correlation tests were performed in selective ROIs where the rates DTI changes were significantly different between PD and HC groups. All tests were two-tailed and a false discovery rate (FDR) corrected value of *q* < 0.05 [22] was used as significance threshold.

## Results

### Clinical, DAT, and CSF biomarker group characteristics

The group demographics and clinical characteristics are summarized in Table 1, listing baseline values as well as annualized rates of change in clinical scores, DAT ratios, and CSF biomarker concentrations. PD patients and controls were similar in age and gender and also had similar DTI scan intervals. As expected, PD patients performed at baseline significantly worse than HC subjects based on clinical measures. Specifically, PD patients performed significantly worse on total UPDRS scores, UPDRS-III, Hoehn-Yahr, and MoCA tests. PD patients also had smaller putaminal DAT binding ratios than HC subjects per definition, because an abnormally small DAT ratio was one of the criteria for PD. In contrast, PD patients and HC subjects did not differ at baseline with regard to CSF biomarker concentration. After one year, PD patients showed significant worsening in total UPDRS, UPDRS-III, Hoehn-Yahr, MOCA, and putaminal DAT measures. When compared to the changes in the HC group, however, only the decline in total UPDRS and Hoehn-Yahr scores survived significance (group comparison of DAT changes are not available because the control group had no one year follow-up DAT scans). In a subset of 50 PD patients and 35 HC subjects whose longitudinal CSF biomarker data was available at the time of this analysis, no significant changes in CSF biomarker concentrations were found.

**Table 1:** Demographics.

			Baseline				Changes per year			
	HC		PD			PD vs. HC	HC	PD	PD vs. HC	
	Baseline	Follow-up	Baseline	Follow-up		*p*	Changes	Changes	Changes	*p*
No. of Subjects	50		122			—	—	—	—	
Age at MRI	60.6±11		60.5±9			0.92	—	—	—	
Sex (% of Male)	64%		65%			0.92	—	—	—	
MRI interval (month)	12.7±1		12.6±1			0.90	—	—	—	
Side of symptom^[1]^	—		51L: 70R: 1Sym			—	—	—	—	
No. of medication^[2]^	—		46 OFF: 76 ON			—	—	—	—	
total UPDRS^[3]^	3.0±3.3	3.9±4.0	30.5±13		35.8±16	**<0.001**	0.9±0.4	**4.8±1.1**	**4.1±1.6**	**0.02**
UPDRS-III^[4]^	0.6±1.5	1.0±1.9	20.4±9		22.4±10	**<0.001**	0.4±0.2	**1.8±0.8**	1.6±1.1	0.17
Hoehn-Yahr^[5]^	0.0±0.0	0.1±0.3	1.6±0.5		1.8±0.5	**<0.001**	0.0±0.0	**0.2±0.0**	**0.2±0.1**	**0.03**
MoCA^[6]^	28.3±1.1	27.3±2.1	27.5±2.1		26.8±2.8	**0.01**	**-0.9±0.3**	**-0.6±0.2**	0.21±0.4	0.59
Putaminal DAT^[7]^*	1.81±0.3	—	0.66±0.3		0.57±0.2	**<0.001**	—	**-0.05±0.01**	—	—
CSF α-synuclein^[8]^^#^	1862±799	1886±742	1860±813		1821±838	0.99	14.9±94	-16.4±62	-44.2±107	0.68
CSF Aβ_1–42_^[^^9^^]^^#^	362±77	392±89	370±85		383±90	0.61	30.4±9	14.4±8.8	-18.1±13	0.18
CSF t-Tau^[10]^^#^	44.0±19	45.8±20	43.5±16		42.4±16	0.99	1.03±1.1	-1.21±0.9	-2.50±1.4	0.07
CSF p-Tau_181_^[11]^^#^	15.7±8	16.1±8	14.8±7		14.7±7	0.62	-0.67±1.8	-0.68±1.3	-0.14±2.2	0.95

^[1]^ Dominant side of symptom at onset. L = left side; R = right side, Sym = symmetrical

^[2]^ At baseline MRI, all 122 PD patients were drug naïve. At one year follow-up, 76 PD patients started taking levodopa medication.

^[3]^ Unified Parkinson’s Disease Rating Scale (Movement Disorder Society revision) part I-IV, total 65 items, each item ranges from 0 (normal) to 4 (severe)

^[4]^ Unified Parkinson’s Disease Rating Scale (Movement Disorder Society revision) part III, 33 items of motor examination, each item ranges from 0 (normal) to 4 (severe)

^[5]^ Hoehn and Yahr scale, range 0 (best) to 5 (worst). All patients had a score ≤ 2 at baseline per enrollment criterion for PD in the PPMI

^[6]^ Montreal Cognitive Assessment, range from 0 (worst) to 30 (best)

^[7]^ Putaminal dopamine transporter binding ratio (the minimum putaminal side at baseline)

^[8]^ Cerebrospinal fluid alpha-synuclein concentration (ng/ml)

^[9]^ Cerebrospinal fluid beta-amyloid 42 concentration (ng/ml)

^[10]^ Cerebrospinal fluid concentration of total Tau protein (ng/ml)

^[11]^ Cerebrospinal fluid concentration of phosphorylated Tau protein at threonine 181 (ng/ml)

* Follow-up DAT scan data from 6 subjects missing

^#^ Longitudinal data of CSF biomarkers from 50 PD patients and 35 HC subjects available only at the time of the analysis

—indicates not applicable

Significant group differences are **bolded**

### Longitudinal changes in regional DTI

**Fig 3:**
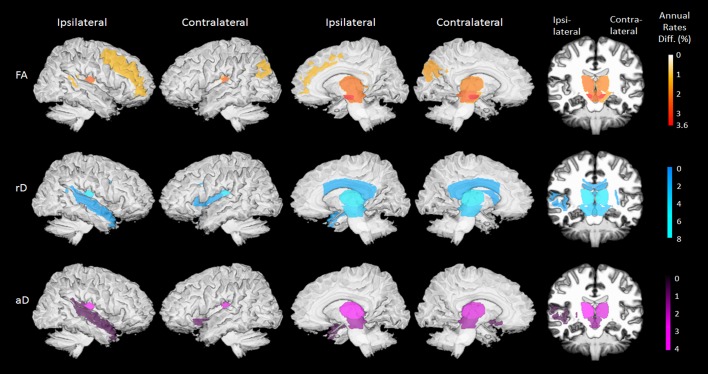
Surface rendered brain maps of differences in regional DTI rates between PD patients and control subjects (FDR-corrected *P*_*FDR*_<0.05). Color scales indicate annual percentage change form baseline, separately for decline in fractional anisotropy (FA), increase in radial diffusivity (rD) and increase in axial diffusivity (aD).

**Fig 4:**
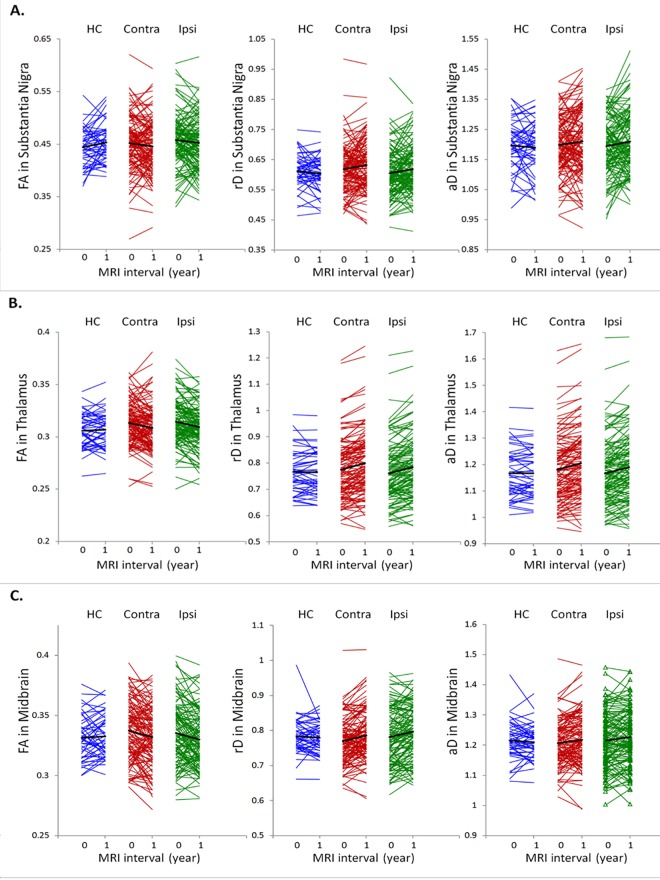
**Individual trajectories of FA, rD and aD changes in the substantia nigra (A), the thalamus (B), and the midbrain (C) from all subjects.** A black line indicates the mean trajectory of each respective group.

Brain regions with significant group differences in annualized DTI rates are displayed in Fig 3, separately for FA, rD or aD measures. A numerical summary of the group differences is given in S1 Table. At baseline, differences in regional DTI between patients and HC subjects were not significant. After one year, PD patients showed steeper DTI changes than HC subjects in several brain regions: Specifically, PD patients showed steeper FA reduction bilaterally in the substantia nigra (ipsilateral: 3.5±1.4%/year, *P*_*FDR*_ = 0.03; contralateral: 3.6±1.4%/year, *P*_*FDR*_ = 0.02), the midbrain (ipsilateral: 2.5±0.8%/year, *P*_*FDR*_ = 0.02; contralateral: 2.3±0.8%/year, *P*_*FDR*_ = 0.02), and the thalamus (ipsilateral: 2.1±0.7%/year, *P*_*FDR*_ = 0.005; contralateral: 1.8±0.7%/year, *P*_*FDR*_ = 0.02). Individual trajectories of the annual FA changes in these regions are depicted in Fig 4. In addition, less prominent FA changes (up to 1.7±0.7%/year, *P*_*FDR*_ = 0.03) in PD patients involved the contralateral cerebral peduncle, the ipsilateral tapatum of the callosum as well as contralateral superior occipital and ipsilateral middle frontal white matter. PD patients also showed a steeper rD increase over time than HC subjects in the bilateral thalamus (ipsilateral: 7.9±2.9%/year, *P*_*FDR*_ = 0.02; contralateral: 8.0±3.1%/year, *P*_*FDR*_ = 0.02), the cerebral peduncle (ipsilateral: 4.9±1.7%/year, *P*_*FDR*_ = 0.007; contralateral: 5.8±1.7%/year, *P*_*FDR*_ = 0.002), the midbrain (ipsilateral: 5.1±2.0%/year, *P*_*FDR*_ = 0.02; contralateral: 5.0±1.9%/year, *P*_*FDR*_ = 0.02), and the substantia nigra (ipsilateral: 4.2±1.7%/year, *P*_*FDR*_ = 0.03; contralateral: 4.0±1.7%/year, *P*_*FDR*_ = 0.04). In addition, less prominent rD changes (up to 3.2±1.3%/year, *P*_*FDR*_ = 0.03) in PD involved the body and splenium of the corpus callosum, the contralateral external capsule and ipsilateral superior temporal white matter. PD patients also had a steeper aD increase over one year than HC subjects bilaterally in the thalamus (ipsilateral and contralateral: 4.0±1.5%/year, *P*_*FDR*_ = 0.01) and to a lesser extent (up to 2.8±1.2%/year, *P*_*FDR*_ = 0.03) in the bilateral midbrain, ipsilateral retrolenticular part of internal capsule, contralateral inferior fronto-occipital fasciculus and in ipsilateral superior temporal white matter. Differences in DTI changes between the ipsilateral and contralateral sides in PD patients were not significant.

### Correlations between clinical and DTI changes

Correlations between clinical progression and DTI changes are summarized in Table 2. No significant correlation was observed between progression in motor dysfunction based on UPDRS scores and DTI changes. In contrast, fast cognitive decline based on MoCA scores correlated with a steep rD as well as aD increase in the bilateral thalamus (ipsilateral: *P*_*FDR*_ = 0.03, *r* = -0.22 for rD and *P*_*FDR*_ = 0.01, *r* = -0.25 for aD; contralateral: *P*_*FDR*_ = 0.001, *r* = -0.30 for rD and *r* = -0.32 for aD).

**Table 2:** Pearson’s correlation results between regional DTI changes and changes in clinical or bio-specimen changes.

Measure	Brain Region	Hemi-sphere	Pearson’s Correlation	Total UPDRS	UPDRS-III	MoCA total	Putaminal DaT^a^	CSF α-synuclein^b^	CSF Aβ_1–42_^b^	CSF t-Tau^b^	CSF p-Tau_181_^b^
FA	Substantia	Ipsi-	Coefficient	0.03	-0.04	0.03	0.12	0.24	0.18	**0.45**	0.04
	Nigra		*(P*_*FDR*_*)*	(*n*.*s*.)	(*n*.*s*.)	(*n*.*s*.)	(*n*.*s*.)	(*n*.*s*.)	(*n*.*s*.)	**(0.001)**	(*n*.*s*.)
		Contra-	Coefficient	-0.05	-0.08	0.01	**0.25**	0.17	-0.08	0.18	-0.05
			*(P*_*FDR*_*)*	(*n*.*s*.)	(*n*.*s*.)	(*n*.*s*.)	**(0.01)**	(*n*.*s*.)	(*n*.*s*.)	(*n*.*s*.)	(*n*.*s*.)
	MidBrain	Ipsi-	Coefficient	-0.05	-0.12	0.20	0.06	0.22	-0.03	0.19	-0.14
			*(P*_*FDR*_*)*	(*n*.*s*.)	(*n*.*s*.)	(0.05)	(*n*.*s*.)	(*n*.*s*.)	(*n*.*s*.)	(*n*.*s*.)	(*n*.*s*.)
		Contra-	Coefficient	-0.07	-0.12	0.10	0.12	0.14	-0.08	0.17	-0.21
			*(P*_*FDR*_*)*	(*n*.*s*.)	(*n*.*s*.)	(*n*.*s*.)	(*n*.*s*.)	(*n*.*s*.)	(*n*.*s*.)	(*n*.*s*.)	(*n*.*s*.)
	Thalamus	Ipsi-	Coefficient	-0.06	-0.09	0.09	0.05	0.00	-0.14	0.19	-0.07
			*(P*_*FDR*_*)*	(*n*.*s*.)	(*n*.*s*.)	(*n*.*s*.)	(*n*.*s*.)	(*n*.*s*.)	(*n*.*s*.)	(*n*.*s*.)	(*n*.*s*.)
		Contra-	Coefficient	-0.11	-0.12	0.10	0.23	0.06	-0.16	0.18	-0.21
			*(P*_*FDR*_*)*	(*n*.*s*.)	(*n*.*s*.)	(*n*.*s*.)	(0.03)	(*n*.*s*.)	(*n*.*s*.)	(*n*.*s*.)	(*n*.*s*.)
rD	Substantia	Ipsi-	Coefficient	-0.03	0.06	0.04	-0.10	-0.34	-0.31	**-0.44**	-0.22
	Nigra		*(P*_*FDR*_*)*	(*n*.*s*.)	(*n*.*s*.)	(*n*.*s*.)	(*n*.*s*.)	(0.03)	(0.06)	**(0.001)**	(*n*.*s*.)
		Contra-	Coefficient	0.04	0.06	0.03	-0.22	-0.15	0.01	-0.14	0.01
			*(P*_*FDR*_*)*	(*n*.*s*.)	(*n*.*s*.)	(*n*.*s*.)	(0.04)	(*n*.*s*.)	(*n*.*s*.)	(*n*.*s*.)	(*n*.*s*.)
	MidBrain	Ipsi-	Coefficient	0.03	0.10	-0.19	-0.01	-0.16	0.08	-0.07	0.10
			*(P*_*FDR*_*)*	(*n*.*s*.)	(*n*.*s*.)	(0.08)	(*n*.*s*.)	(*n*.*s*.)	(*n*.*s*.)	(*n*.*s*.)	(*n*.*s*.)
		Contra-	Coefficient	0.08	0.11	-0.17	-0.05	-0.15	0.22	-0.08	0.22
			*(P*_*FDR*_*)*	(*n*.*s*.)	(*n*.*s*.)	(*n*.*s*.)	(*n*.*s*.)	(*n*.*s*.)	(*n*.*s*.)	(*n*.*s*.)	(*n*.*s*.)
	Thalamus	Ipsi-	Coefficient	0.05	0.11	-0.22	-0.09	-0.10	0.20	-0.05	0.15
			*(P*_*FDR*_*)*	(*n*.*s*.)	(*n*.*s*.)	(0.03)	(*n*.*s*.)	(*n*.*s*.)	(*n*.*s*.)	(*n*.*s*.)	(*n*.*s*.)
		Contra-	Coefficient	0.10	0.10	**-0.30**	-0.08	-0.13	0.18	-0.03	0.20
			*(P*_*FDR*_*)*	(*n*.*s*.)	(*n*.*s*.)	**(0.001)**	(*n*.*s*.)	(*n*.*s*.)	(*n*.*s*.)	(*n*.*s*.)	(*n*.*s*.)
aD	Substantia	Ipsi-	Coefficient	-0.05	-0.01	0.06	-0.09	-0.33	-0.21	-0.28	-0.27
	Nigra		*(P*_*FDR*_*)*	(*n*.*s*.)	(*n*.*s*.)	(*n*.*s*.)	(*n*.*s*.)	(0.03)	(*n*.*s*.)	(*n*.*s*.)	(*n*.*s*.)
		Contra-	Coefficient	-0.04	-0.04	0.04	-0.13	-0.02	-0.06	-0.09	-0.06
			*(P*_*FDR*_*)*	(*n*.*s*.)	(*n*.*s*.)	(0.66)	(*n*.*s*.)	(*n*.*s*.)	(*n*.*s*.)	(*n*.*s*.)	(*n*.*s*.)
	MidBrain	Ipsi-	Coefficient	0.01	0.05	-0.14	-0.01	-0.07	0.13	-0.01	0.09
			*(P*_*FDR*_*)*	(*n*.*s*.)	(*n*.*s*.)	(0.13)	(*n*.*s*.)	(*n*.*s*.)	(*n*.*s*.)	(*n*.*s*.)	(*n*.*s*.)
		Contra-	Coefficient	0.03	0.02	-0.18	-0.04	-0.09	0.25	0.01	0.21
			*(P*_*FDR*_*)*	(*n*.*s*.)	(*n*.*s*.)	(0.04)	(*n*.*s*.)	(*n*.*s*.)	(*n*.*s*.)	(*n*.*s*.)	(*n*.*s*.)
	Thalamus	Ipsi-	Coefficient	0.05	0.10	**-0.25**	-0.17	-0.11	0.19	-0.03	0.15
			*(P*_*FDR*_*)*	(*n*.*s*.)	(*n*.*s*.)	**(0.01)**	(*n*.*s*.)	(*n*.*s*.)	(*n*.*s*.)	(*n*.*s*.)	(*n*.*s*.)
		Contra-	Coefficient	0.08	0.08	**-0.32**	-0.05	-0.11	0.19	0.02	0.15
			*(P*_*FDR*_*)*	(*n*.*s*.)	(*n*.*s*.)	**(0.001)**	(*n*.*s*.)	(*n*.*s*.)	(*n*.*s*.)	(*n*.*s*.)	(*n*.*s*.)

Listed are Pearson correlation coefficient and p-value in brackets

^a^ DAT data from 6 subjects were missing and are not included

^b^ Data of CSF biomarker concentration changes were available from a subset of 50 PD patients at the time of the analysis

**Bold**: Correlations are also significant on the Spearman rank correlation test.

### Correlations between putaminal DAT and DTI changes

Correlation between changes in putaminal DAT binding and changes in regional DTI is shown in Table 2. Growing dopamine deficiency based on putaminal DAT binding ratio correlated with a steep FA reduction (*P*_*FDR*_ = 0.01, *r* = 0.25) and a steep rD increase (*P*_*FDR*_ = 0.04, *r* = -0.22) in the contralateral substantial nigra. Growing dopamine deficiency also correlated with FA reduction (*P*_*FDR*_ = 0.03, *r* = 0.23) in the contralateral thalamus.

### Correlations between CSF-biomarker and DTI changes

**Fig 5:**
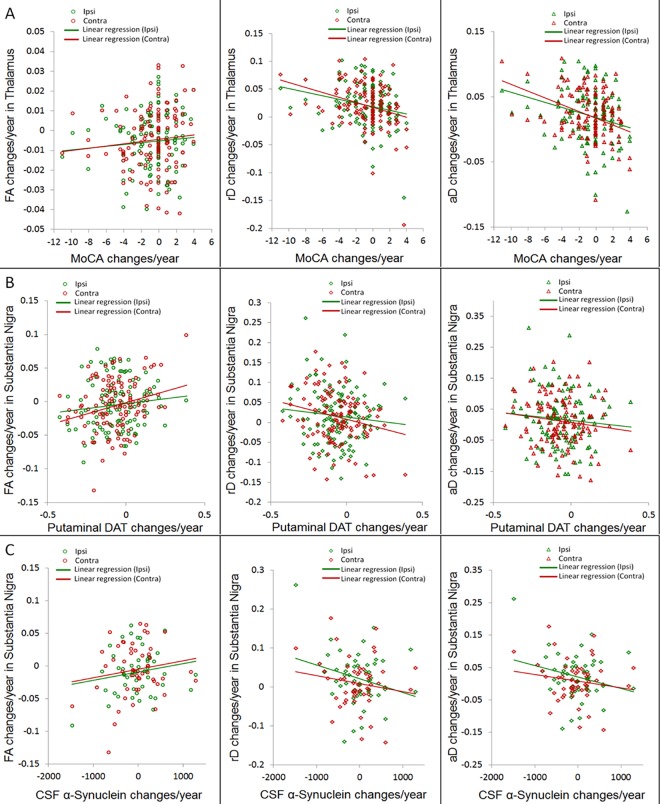
**Scatter plots of the most prominent correlations:** (A), correlation between annual DTI changes in the thalamus and MoCA score changes, (B) correlation between annual DTI changes in the substantia nigra and changes of putaminal DAT binding ratios (the putaminal side with minimum DAT ratio at baseline was selected). (C), correlation between annual DTI changes in the substantia nigra and changes of CSF alpha-synuclein in a subset of 50 PD patients.

Correlations between changes in CSF bio-marker concentrations and DTI are also listed in Table 2. Based on a subset of 50 PD patients who had longitudinal CSF biomarker data, more decrease in CSF α-synuclein concentration over time correlated with a steep increase of rD (*P*_*FDR*_ = 0.03, *r* = -0.34) as well as aD (*P*_*FDR*_ = 0.03, *r* = -0.33) in the ipsilateral substantia nigra. Similarly, more decrease in CSF total-Tau concentration correlated with a steep FA reduction (*P*_*FDR*_ = 0.001, *r* = 0.45) as well as a steep rD increase (*P*_*FDR*_ = 0.001, *r* = -0.44) in the ipsilateral substantia nigra. Scatter plots of the most prominent correlation findings are illustrated in Fig 5.

## Discussion

The main finding of this study is that PD is associated with higher than normal rates of regional DTI changes that involve primarily the substantia nigra, the thalamus and midbrain regions. This regional pattern of DTI changes is consistent with neuropathological reports of distributed neurodegeneration in PD [23]. In addition, the DTI changes in PD, particularly from the substantia nigra, positively correlated with growing dopamine deficiency as well as with progressive reduction of a-synuclein in CSF. These findings imply that DTI changes reflect–at least in part–progression of PD pathology. Taken together, the current results and similar results from two other studies [7,8] demonstrate that diffusion MRI change in the substantia nigra is a valuable biomarker of PD progression.

The finding of higher than normal DTI rates, especially FA and rD, in the substantia nigra, thalamus and midbrain is consistent with neuropathological reports [23,24] that indicate a high vulnerability of these brain regions to PD. The regional distribution of DTI changes is also consistent with the pathological stage III of the sporadic PD progression [23], when Lewy bodies and Lewy neurites lesions are involved in midbrain, in particular in the pars compacta of the substantia nigra. The finding of abnormal DTI changes in thalamus is also not surprising. Similar to our finding of abnormal DTI changes in the thalamus over time, another study reported FA reduction along the thalamic projection fiber in de novo PD patients [25]. A key neuropathological feature of PD is that the loss of dopamine neurons in the substantia nigra and the midbrain further leads to a degeneration of the nigro-striatal and basal ganglia-thalamocortical pathways, ultimately impacting the thalamus [26]. The Braak’s stage IV of PD progression also involves neuropathological lesions in the thalamus [23].

It is also interesting to note that abnormal DTI changes occurred in regions of deep association tracts such as the splenium and tapatum of the corpus callosum, the external capsule, cerebral peduncle, retrolenticular internal capsule, inferior frontooccipital fasciculus, and white matter regions underlying the middle frontal, superior occipital and superior temporal neocortices. According to Braak’s staging of PD, Lewy body pathology progresses to mesocortex and neocortices at later disease stages IV to VI. Whether the abnormal DTI changes in cortico-cortical pathways are predictive for advanced PD progression warrants further studies.

This study found a strong positive correlation between increased changes in thalamic diffusivities (rD and aD) and progressive cognitive decline in PD patients. The thalamus is known for its role in cognitive behavior, including learning and memory, inhibitory control, decision-making, and the control of visual orienting response [27–30]. A longitudinal structural MRI study in PD [31] also indicated increased thalamic atrophy is associated with a decline in cognitive function. The DTI results from this study suggest that microstructural changes in the thalamus might be an indicator of cognitive decline in PD. Whether changes in thalamic DTI is a progression marker of cognitive decline needs to be studied further with longer follow-up times of imaging data and more detailed cognitive measurements.

The finding of relationships between high DTI changes in the substantia nigra and increasing putaminal DAT deficiency in PD is novel. This finding links declining nigral integrity to striatal dopamine transporter deficiency, potentially via the nigrostriatal dopaminergic pathway. The finding is also in line with previous cross-sectional DTI results from PPMI subjects [18] and with studies of dopaminergic degeneration of the substantia nigra in animal models [32]. Considering that changes in the DAT system may be unstably and altered by drug treatment, DTI measurements of changes in microstructural integrity might provide an alternative marker for disease modifying interventions in PD.

The positive correlation between increased nigral diffusivity (rD and aD) over time and diminished CSF α-synuclein concentration in a subsample of PD patients is also worth to discuss. Low α-synuclein in CSF has been proposed as a biomarker candidate for PD as the protein accumulates in Lewy bodies and Lewy neurites of the brain parenchyma [15,17,33–35]. This raises the possibility that DTI findings in PD of abnormal diffusivity in the substantia nigra indicate increasing α-synuclein accumulation in the brain. However, this observation was still statistically weak and might be driven by outliers. The finding needs to be validated by further studies with sufficient longitudinal collections of CSF biomarkers. Also interesting in this context is the finding of a positive correlation between increased changes in nigral DTI and higher CSF total tau concentration in this PD subset. Elevated CSF total tau protein has consistently been found in patients with Alzheimer’s disease [36–38]. Elevated CSF total tau has also been suggested as indicator of axonal degeneration [39–41]. It is conceivable that the correlation between changes in nigral DTI and CSF total tau indicates comorbid Alzheimer’s pathology in this group of PD patients. Alternatively, the finding might indicate an intriguing interaction between a-synuclein and tau pathology that warrant further studies.

An interpretation of the DTI alterations in terms of their underlying microstructural origins is notoriously difficult, because DTI is sensitive to a broad spectrum of microscopic alterations in brain tissue, and the model-based concept of DTI has theoretical limitations that can mislead when the model is violated, such as in case of crossing-fiber bundles. In general, however, an increase in rD has been associated with demyelination whereas an increase in aD is thought to primarily indicate cell degradation and loss. Either of these changes theoretically leads to reduced FA. Although for most of the brain regions, decreased FA and increased diffusivity (rD and aD) occurred consistently together, in some regions FA changes dominated rD or aD changes and vice versa. We cautiously interpret these differences as index of the variability in microstructural alterations in PD. In addition, because of fundamental limitations in DTI, the degree to which the changes in gray matter areas reflect degeneration of passing fibers or of local gray matter remains unclear. This distinction can only be made with more technically advanced MRI methods, such as Q-space [42] or diffusion spectrum imaging [43].

The study has several merits: First, this DTI study replicates two previous reports of progressive microstructural changes in the substantia nigra in PD on a larger sample across multiple MRI centers. The finding emphasizes that diffusion MRI of the substantia nigra is an important marker in assessing PD progression. Second, the study indicates that progressive microstructural degeneration beyond the substantia nigra, encompassing mainly the thalamus and the midbrain, occurs in early stages of PD. Third, the novel finding that microstructural degeneration in the substantia nigra is correlated with increasing dopaminergic deficits as well as abnormal CSF biomarker concentrations in PD implies that DTI can be useful for monitoring the course of the disease and also for assessing efficacy of treatment interventions. Lastly, the study demonstrates the practicability of multi-center diffusion imaging in PD.

Several limitations of our study should be mentioned. The clinical diagnosis of PD was not confirmed by autopsy. Aside from diagnostic uncertainty, it is possible that some patients diagnosed with PD also had other comorbid neurological conditions that may have skewed the outcome of this study. Another limitation is that some patients went on PD medication during the first year of the study, which may have increased the variability in symptomatology across patients and thus reduced sensitivity to detect correlations between DTI and clinical changes. A statistical limitation is that contrary to a FDR corrected statistical threshold of p = 0.025, a Bonferroni correction for multiple comparison yielded no significant results. We can conclude with high confidence (97.5%) that DTI shows an effect of microstructural progression in PD, although we cannot say that any single significant result is accurate. Lastly, results of correlations between changes in CSF-biomarkers and DTI may be biased because there was no control of the subset of PD patients with longitudinal CSF biomarker data.

## Supporting Information

S1 File Appendix. Complete list of PPMI authors and study investigators.
(DOCX)

S1 Table Group DTI values at baseline and one year follow-up as well as estimates of annual changes, separately listed by brain region. (DOCX)
