# Supplementary material for: Progression of Regional Microstructural Degeneration in Parkinson’s Disease: A Multicenter Diffusion Tensor Imaging Study
Source: PLoS One. 2016 Oct 31;11(10):e0165540. doi: 10.1371/journal.pone.0165540 (PMC5087900; doi:10.1371/journal.pone.0165540)
Supplement: S1 File — Complete list of PPMI authors and study investigators. (DOCX) [file pone.0165540.s001.docx]

**S1 File. Appendix.**

## Complete list of PPMI authors and study investigators:

#### **PPMI Steering Committee:** Kenneth Marek, MD^1^ (Principal Investigator); Danna Jennings, MD^1^ (Olfactory  Core, PI; Site Investigator); Shirley Lasch, MBA^1^; Caroline Tanner, MD, PhD^9^ (Site Investigator);  Tanya Simuni, MD^3^ (Site Investigator); Christopher Coffey, PhD^4^ (Statistics Core, PI); Karl Kieburtz, MD, MPH^5^ (Clinical Core, PI); Renee Wilson^5^; Werner Poewe, MD^7^ (Site Investigator); Brit Mollenhauer, MD^8^ (Bioanalytics Core, co-PI; Site Investigator); Douglas Galasko, MD^27^ (Bioanalytics Core, co-PI; Site Investigator); Tatiana Foroud, PhD^15^ (Genetics Coordination Core and Biorepository, PI); Todd Sherer, PhD^6^; Sohini Chowdhury^6^; Mark Frasier, PhD^6^; Catherine Kopil, PhD^6^; Vanessa Arnedo^6^

#### **PPMI Study Cores:** *Clinical Coordination Core*: Alice Rudolph, PhD^5^; Cynthia Casaceli, MBA^5^. *Imaging Core*: John Seibyl, MD^1^ (Principal Investigator); Susan Mendick, MPH^1^; Norbert Schuff, PhD^9^. *Statistics Core*: Chelsea Caspell^4^; Liz Uribe^4^; Eric Foster ^4^; Katherine Gloer PhD^4^; Jon Yankey MS^4^. *Bioinformatics Core*: Arthur Toga, PhD^10^ (Principal Investigator); Karen Crawford^10^. *Biorepository*: Paola Casalin^11^; Giulia Malferrari^11^. *Genetics Core*: Andrew Singleton, PhD^13^ (Principal Investigator). *Neuropsychological and Cognitive Assessments*: Keith A.  Hawkins, PsyD^14^

#### **PPMI Investigators:** David Russell, MD, PhD^1^; Stewart Factor, DO^16^; Penelope Hogarth, MD^17^; David Standaert, MD, PhD^18^; Robert Hauser, MD, MBA^19^; Joseph Jankovic, MD^20^; Matthew Stern, MD^12^; Lama Chahine, MD^12^; James Leverenz, MD^21^; Samuel Frank, MD^22^; Irene Richard, MD^23^;  Klaus Seppi, MD^7^; Holly Shill, MD^24^; Hubert Fernandez, MD^25^; Daniela Berg, MD^26^; Isabel Wurster MD^26^; Zoltan Mari, MD^28^; David Brooks, MD^29^; Nicola Pavese, MD^29^; Paolo Barone, MD, PhD^30^; Stuart Isaacson, MD^31^; Alberto Espay, MD, MSc^32^; Dominic Rowe, MD, PhD^33^; Melanie Brandabur MD^2^; James Tetrud MD^2^; Grace Liang MD^2^; Alex Iranzo, MD^34^; Eduardo Tolosa MD^34^; Shu-Ching Hu, MD, PhD^21^; Gretchen Todd^21^.

**PPMI Coordinators:** Laura Leary^1^; Cheryl Riordan^1^;  Linda Rees, MPH^2^; Alicia Portillo^17^; Art Lenahan^17^; Karen Williams^3^; Stephanie Guthrie, MSN^18^; Ashlee Rawlins^18^; Sherry Harlan^19^; Christine Hunter, RN^20^; Baochan Tran^12^; Abigail Darin^12^; Carly Linder^12^; Marne Baca^21^; Heli Venkov^21^; Cathi-Ann Thomas, RN, MS^22^; Raymond James, RN^22^; Cheryl Deeley, MSN^23^; Courtney Bishop BS^23^; Fabienne Sprenger, MD^7^; Diana Willeke^8^; Sanja Obradov^24^; Jennifer Mule^25^; Nancy Monahan^25^; Katharina Gauss^26^; Deborah Fontaine, BSN, MS^27^; Christina Gigliotti^27^; Arita McCoy^28^; Becky Dunlop^28^; Bina Shah, BSc^29^; Susan Ainscough^30^; Angela James^31^; Rebecca Silverstein^31^; Kristy Espay^32^; Madelaine Ranola^33^

#### **SAB (Industry Scientific Advisory Board):** Thomas Comery, PhD^35^; Jesse Cedarbaum, MD^36^; Bernard Ravina, MD, MSCE^36^; Igor D. Grachev, MD, PhD^37^; Jordan S. Dubow, MD^38^; Michael Ahlijanian, PhD^39^; Holly Soares, PhD^39^; Suzanne Ostrowizki, MD, PhD^40^; Paulo Fontoura, MD, PhD^40^; Alison Chalker, PhD^41^; David L. Hewitt, MD^41^; Marcel van der Brug, PhD^42^; Alastair D. Reith, PhD^43^; Peggy Taylor, ScD^44^; Jan Egebjerg, PhD^45^; Mark Minton, MD^46^; Andrew Siderowf, MD, MSCE^46^; Pierandrea Muglia, PhD^47^; Robert Umek, PhD^48^; Ana Catafau, MD,PhD^48^; Vera Kiyasova, MD, PhD^50^; Barbara Saba^50^

**PPMI Group Affiliation:**

^1^ Institute for Neurodegenerative Disorders, New Haven, CT.

^2^ The Parkinson’s Institute, Sunnyvale, CA.

^3^ Northwestern University, Chicago, IL.

^4^ University of Iowa, Iowa City, IA.

^5^ Clinical Trials Coordination Center, University of Rochester, Rochester, NY.

^6^ The Michael J. Fox Foundation for Parkinson’s Research, New York, NY.

^7^ Innsbruck Medical University, Innsbruck, Austria.

^8^ Paracelsus-Elena Klinik, Kassel, Germany.

^9^ University of California, San Francisco, CA.

^10^ Laboratory of Neuroimaging (LONI), University of Southern California.

^11^ BioRep, Milan, Italy.

^12^ University of Pennsylvania, Philadelphia, PA.

^13^ National Institute on Aging, NIH, Bethesda, MD.

^14^ Yale University, New Haven, CT.

^15^ Indiana University, Indianapolis, IN.

^16^ Emory University of Medicine, Atlanta, GA.

^17^ Oregon Health and Science University, Portland, OR.

^18^ University of Alabama at Birmingham, Birmingham, AL.

^19^ University of South Florida, Tampa, FL.

^20^ Baylor College of Medicine, Houston, TX.

^21^ University of Washington, Seattle, WA.

^22^ Boston University, Boston, MA.

^23^ University of Rochester, Rochester, NY.

^24^ Banner Research Institute, Sun City, AZ.

^25^ Cleveland Clinic, Cleveland, OH.

^26^ University of Tuebingen, Tuebingen, Germany.

^27^ University of California, San Diego, CA.

^28^ Johns Hopkins University, Baltimore, MD.

^29^ Imperial College of London, London, UK.

^30^ University of Salerno, Salerno, Italy.

^31^ Parkinson’s Disease and Movement Disorders Center, Boca Raton, FL.

^32^ University of Cincinnati, Cincinnati, OH.

^33^ Macquarie University, Sydney Australia.

^34^ Hospital Clinic of Barcelona, Barcelona, Spain.

^35^ Pfizer, Inc., Groton, CT.

^36^ Biogen Idec, Cambridge, MA.

^37^ GE Healthcare, Princeton, NJ.

^38^ AbbVie, Abbot Park, IL.

^39^ Bristol-Myers Squibb Company.

^40^ F.Hoffmann La-Roche, Basel, Switzerland.

^41^ Merck & Co., North Wales, PA.

^42^ Genentech, Inc., South San Francisco, CA.

^43^ GlaxoSmithKline, Stevenage, United Kingdom.

^44^ Covance, Dedham, MA.

^45^ H. Lundbeck A/S.

^46^ Avid Radiopharmaceuticals, Philadelphia , PA.

^47^ UCB Pharma S.A., Brussels, Belgium.

^48^ Meso Scale Discovery.

^49^ Piramal Life Sciences, Berlin, Germany.

^50^ Servier.
