# Supplementary material for: Progression of Regional Microstructural Degeneration in Parkinson’s Disease: A Multicenter Diffusion Tensor Imaging Study
Source: PLoS One. 2016 Oct 31;11(10):e0165540. doi: 10.1371/journal.pone.0165540 (PMC5087900; doi:10.1371/journal.pone.0165540)
Supplement: S1 Table — (DOCX) [file pone.0165540.s002.docx]

**S1 Table. Group DTI values at baseline and one year follow-up as well as estimates of annual changes, separately listed by brain region.**

| Region of Interest | Hemi-sphere | Measure | Baseline | |  | Follow-up | |  | Estimated annual rates from baseline | | | | |  |
| --- | --- | --- | --- | --- | --- | --- | --- | --- | --- | --- | --- | --- | --- | --- |
|  |  |  | HC | PD |  | HC | PD |  | HC | PD |  | PD *vs.* HC | *P*_FDR_ | |
| Substantia | Ipsi- | FA | 0.441±0.04 | 0.456±0.05 |  | 0.452±0.04 | 0.451±0.05 |  | 2.40±1.1 | -1.11±0.8 |  | **-3.54±1.4** | **0.03** | |
| Nigra |  | rD | 0.616±0.07 | 0.605±0.08 |  | 0.604±0.06 | 0.621±0.08 |  | -1.90±1.2 | 2.39±1.0 |  | **4.20±1.7** | **0.03** | |
|  |  | aD | 1.184±0.11 | 1.192±0.10 |  | 1.190±0.08 | 1.213±0.10 |  | 0.39±1.2 | 1.50±0.7 |  | 1.20±1.3 | *n.s.* | |
|  | Contra- | FA | 0.441±0.04 | 0.452±0.05 |  | 0.452±0.04 | 0.446±0.05 |  | 2.40±1.1 | -1.17±0.8 |  | **-3.61±1.4** | **0.02** | |
|  |  | rD | 0.616±0.07 | 0.619±0.08 |  | 0.604±0.06 | 0.632±0.08 |  | -1.90±1.2 | 1.99±1.0 |  | **3.97±1.7** | **0.04** | |
|  |  | aD | 1.184±0.11 | 1.199±0.10 |  | 1.190±0.08 | 1.213±0.11 |  | 0.39±1.2 | 0.90±0.6 |  | 0.60±1.2 | *n.s.* | |
| Midbrain | Ipsi- | FA | 0.331±0.02 | 0.335±0.03 |  | 0.332±0.02 | 0.330±0.02 |  | 0.55±0.7 | -1.57±0.5 |  | **-2.50±0.8** | **0.02** | |
|  |  | rD | 0.804±0.15 | 0.781±0.08 |  | 0.780±0.04 | 0.797±0.08 |  | -3.54±2.9 | 1.88±0.6 |  | **5.15±2.0** | **0.02** | |
|  |  | aD | 1.232±0.14 | 1.214±0.09 |  | 1.210±0.05 | 1.227±0.09 |  | -1.94±1.6 | 0.84±0.4 |  | **2.76±1.2** | **0.04** | |
|  | Contra- | FA | 0.331±0.02 | 0.338±0.03 |  | 0.332±0.02 | 0.333±0.03 |  | 0.55±0.7 | -1.80±0.5 |  | **-2.25±0.8** | **0.02** | |
|  |  | rD | 0.804±0.15 | 0.770±0.06 |  | 0.780±0.04 | 0.786±0.07 |  | -3.54±2.8 | 1.95±0.5 |  | **5.03±1.9** | **0.02** | |
|  |  | aD | 1.232±0.14 | 1.205±0.07 |  | 1.210±0.05 | 1.218±0.08 |  | -1.94±1.6 | 0.93±0.4 |  | **2.80±1.2** | **0.03** | |
| Thalamus | Ipsi- | FA | 0.305±0.02 | 0.315±0.02 |  | 0.307±0.02 | 0.309±0.02 |  | 0.37±0.5 | -1.49±0.4 |  | **-2.06±0.7** | **0.005** | |
|  |  | rD | 0.791±0.16 | 0.762±0.11 |  | 0.773±0.10 | 0.784±0.12 |  | -4.02±3.9 | 3.84±0.9 |  | **7.85±2.9** | **0.02** | |
|  |  | aD | 1.190±0.15 | 1.167±0.12 |  | 1.176±0.11 | 1.189±0.13 |  | -2.01±1.9 | -1.92±0.5 |  | **4.00±1.5** | **0.01** | |
|  | Contra- | FA | 0.305±0.02 | 0.314±0.02 |  | 0.307±0.02 | 0.309±0.02 |  | 0.37±0.5 | -1.29±0.4 |  | **-1.84±0.7** | **0.02** | |
|  |  | rD | 0.791±0.16 | 0.777±0.12 |  | 0.773±0.10 | 0.799±0.12 |  | -4.02±3.9 | 3.66±1.0 |  | **7.95±3.1** | **0.02** | |
|  |  | aD | 1.190±0.15 | 1.183±0.12 |  | 1.176±0.11 | 1.204±0.13 |  | -2.01±1.9 | 1.80±0.5 |  | **3.98±1.5** | **0.02** | |
| Cerebral | Ipsi- | FA | 0.574±0.03 | 0.588±0.03 |  | 0.575±0.03 | 0.581±0.03 |  | 0.38±0.5 | -0.98±0.4 |  | -1.38±0.6 | *n.s.* | |
| Peduncle |  | rD | 0.560±0.08 | 0.534±0.04 |  | 0.546±0.07 | 0.546±0.04 |  | -2.84±2.0 | 2.32±0.8 |  | **4.87±1.7** | **0.006** | |
|  |  | aD | 1.417±0.08 | 1.418±0.06 |  | 1.411±0.07 | 1.426±0.07 |  | -0.47±0.6 | 0.46±0.3 |  | 0.87±0.7 | *n.s.* | |
|  | Contra- | FA | 0.574±0.03 | 0.589±0.03 |  | 0.575±0.03 | 0.582±0.03 |  | 0.38±0.5 | -1.02±0.3 |  | **-1.49±0.6** | **0.03** | |
|  |  | rD | 0.560±0.08 | 0.535±0.05 |  | 0.546±0.07 | 0.551±0.05 |  | -2.84±2.0 | 2.97±0.8 |  | **5.76±1.7** | **0.002** | |
|  |  | aD | 1.417±0.08 | 1.415±0.07 |  | 1.411±0.07 | 1.427±0.08 |  | -0.47±0.6 | 0.68±0.4 |  | 1.20±0.7 | *n.s.* | |
| External | Ipsi- | FA | 0.354±0.02 | 0.363±0.03 |  | 0.356±0.02 | 0.360±0.02 |  | 0.51±0.5 | -0.56±0.4 |  | -1.07±0.7 | *n.s.* | |
| Capsule |  | rD | 0.610±0.04 | 0.598±0.05 |  | 0.604±0.04 | 0.605±0.04 |  | -1.09±0.8 | 0.90±0.5 |  | 1.98±1.0 | *n.s.* | |
|  |  | aD | 1.047±0.05 | 1.049±0.05 |  | 1.046±0.05 | 1.057±0.06 |  | -0.23±0.5 | 0.53±0.3 |  | 0.75±0.6 | *n.s.* | |
|  | Contra- | FA | 0.354±0.02 | 0.362±0.03 |  | 0.356±0.02 | 0.359±0.03 |  | 0.51±0.5 | -0.73±0.5 |  | -1.35±0.8 | *n.s.* | |
|  |  | rD | 0.610±0.04 | 0.594±0.04 |  | 0.604±0.04 | 0.603±0.05 |  | -1.09±0.8 | 1.35±0.6 |  | **2.56±1.0** | **0.03** | |
|  |  | aD | 1.047±0.05 | 1.041±0.05 |  | 1.046±0.05 | 1.050±0.06 |  | -0.23±0.5 | 0.70±0.3 |  | 0.99±0.6 | *n.s.* | |
| Retro- | Ipsi- | FA | 0.512±0.03 | 0.518±0.03 |  | 0.512±0.03 | 0.511±0.03 |  | 0.02±0.5 | -1.06±0.4 |  | -1.09±0.7 | *n.s.* | |
| lenticular |  | rD | 0.523±0.09 | 0.502±0.06 |  | 0.507±0.04 | 0.513±0.06 |  | -3.02±2.7 | 2.01±0.9 |  | 4.78±2.2 | *n.s.* | |
| part of |  | aD | 1.190±0.06 | 1.178±0.07 |  | 1.180±0.05 | 1.190±0.07 |  | -0.70±0.5 | 0.88±0.4 |  | **1.70±0.7** | **0.02** | |
| Internal | Contra- | FA | 0.512±0.03 | 0.523±0.04 |  | 0.512±0.03 | 0.518±0.04 |  | 0.02±0.5 | -0.76±0.3 |  | -0.89±0.6 | *n.s.* | |
| Capsule |  | rD | 0.523±0.09 | 0.492±0.05 |  | 0.507±0.04 | 0.501±0.06 |  | -3.02±2.7 | 1.57±0.6 |  | 4.31±1.9 | *n.s.* | |
|  |  | aD | 1.190±0.06 | 1.175±0.06 |  | 1.180±0.05 | 1.183±0.07 |  | -0.70±0.5 | 0.58±0.4 |  | 1.30±0.7 | *n.s.* | |
| Inferior | Ipsi- | FA | 0.404±0.02 | 0.413±0.03 |  | 0.404±0.02 | 0.409±0.03 |  | 0.05±0.4 | -0.78±0.4 |  | -0.78±0.6 | *n.s.* | |
| Fronto- |  | rD | 0.595±0.04 | 0.588±0.05 |  | 0.592±0.04 | 0.589±0.04 |  | -0.73±0.7 | -0.07±0.6 |  | 0.57±1.0 | *n.s.* | |
| Occipital |  | aD | 1.154±0.05 | 1.160±0.05 |  | 1.148±0.05 | 1.156±0.06 |  | -0.79±0.4 | -0.55±0.4 |  | 0.22±0.6 | *n.s.* | |
| Fasciculus | Contra- | FA | 0.404±0.02 | 0.415±0.03 |  | 0.404±0.02 | 0.412±0.03 |  | 0.05±0.4 | -0.61±0.4 |  | -0.69±0.7 | *n.s.* | |
|  |  | rD | 0.595±0.04 | 0.583±0.04 |  | 0.592±0.04 | 0.590±0.04 |  | -0.73±0.7 | 0.95±0.6 |  | 1.75±1.0 | *n.s.* | |
|  |  | aD | 1.154±0.05 | 1.154±0.06 |  | 1.148±0.05 | 1.163±0.06 |  | -0.79±0.4 | 0.63±0.4 |  | **1.54±0.6** | **0.03** | |
| Body of | — | FA | 0.531±0.04 | 0.532±0.04 |  | 0.527±0.04 | 0.525±0.04 |  | -0.30±0.3 | -0.88±0.2 |  | -0.62±0.4 | *n.s.* | |
| Callosum |  | rD | 0.747±0.10 | 0.743±0.10 |  | 0.749±0.10 | 0.761±0.10 |  | -0.49±1.3 | 2.63±0.7 |  | **2.99±1.2** | **0.03** | |
|  |  | aD | 1.736±0.10 | 1.747±0.10 |  | 1.747±0.10 | 1.759±0.09 |  | 0.37±0.9 | 0.45±0.3 |  | 0.10±0.7 | *n.s.* | |
| Splenium | — | FA | 0.599±0.03 | 0.602±0.03 |  | 0.598±0.03 | 0.596±0.03 |  | -0.12±0.3 | -0.77±0.2 |  | **-0.77±0.3** | **0.04** | |
| of Callosum |  | rD | 0.600±0.07 | 0.594±0.08 |  | 0.597±0.07 | 0.608±0.08 |  | -0.77±1.4 | 2.34±0.7 |  | **3.21±1.3** | **0.03** | |
|  |  | aD | 1.625±0.11 | 1.637±0.09 |  | 1.639±0.10 | 1.650±0.09 |  | 0.64±1.0 | 0.57±0.3 |  | 0.02±0.7 | *n.s.* | |
| Tapatum of | Ipsi- | FA | 0.407±0.03 | 0.412±0.04 |  | 0.405±0.03 | 0.402±0.04 |  | -0.48±0.4 | -2.10±0.4 |  | **-1.70±0.7** | **0.04** | |
| Callosum |  | rD | 1.000±0.13 | 0.992±0.17 |  | 1.021±0.12 | 1.019±0.14 |  | 2.09±1.9 | 2.77±1.5 |  | 0.72±2.5 | *n.s.* | |
|  |  | aD | 1.805±0.21 | 1.793±0.20 |  | 1.837±0.16 | 1.822±0.20 |  | 1.63±1.9 | 1.38±0.7 |  | 0.02±1.6 | *n.s.* | |
|  | Contra- | FA | 0.407±0.03 | 0.401±0.04 |  | 0.405±0.03 | 0.393±0.04 |  | -0.48±0.4 | -1.66±0.4 |  | -1.40±0.7 | *n.s.* | |
|  |  | rD | 1.000±0.13 | 1.014±0.14 |  | 1.021±0.12 | 1.043±0.15 |  | 2.09±1.9 | 3.37±1.0 |  | 1.41±1.9 | *n.s.* | |
|  |  | aD | 1.805±0.21 | 1.802±0.18 |  | 1.837±0.16 | 1.832±0.20 |  | 1.63±1.9 | 1.56±0.6 |  | 0.10±1.6 | *n.s.* | |
| Middle | Ipsi- | FA | 0.330±0.02 | 0.333±0.02 |  | 0.329±0.02 | 0.328±0.02 |  | -0.12±0.4 | -1.00±0.2 |  | **-1.07±0.5** | **0.04** | |
| Frontal |  | rD | 0.613±0.06 | 0.605±0.04 |  | 0.605±0.04 | 0.610±0.04 |  | -1.65±1.1 | 0.38±0.5 |  | 2.12±1.0 | *n.s.* | |
| WM |  | aD | 1.003±0.05 | 0.998±0.04 |  | 0.992±0.04 | 0.999±0.04 |  | -1.18±0.5 | -0.16±0.2 |  | 0.96±0.4 | *n.s.* | |
|  | Contra- | FA | 0.330±0.02 | 0.334±0.02 |  | 0.329±0.02 | 0.329±0.02 |  | -0.12±0.4 | -0.97±0.3 |  | -1.00±0.5 | *n.s.* | |
|  |  | rD | 0.613±0.06 | 0.605±0.04 |  | 0.605±0.04 | 0.611±0.04 |  | -1.65±1.1 | 0.51±0.6 |  | 2.34±1.1 | *n.s.* | |
|  |  | aD | 1.003±0.05 | 0.998±0.04 |  | 0.992±0.04 | 1.000±0.04 |  | -1.18±0.5 | -0.06±0.3 |  | 1.17±0.5 | *n.s.* | |
| Superior | Ipsi- | FA | 0.377±0.03 | 0.383±0.03 |  | 0.379±0.03 | 0.379±0.03 |  | 0.59±0.4 | -0.88±0.4 |  | -1.46±0.7 | *n.s.* | |
| Occipital |  | rD | 0.586±0.05 | 0.573±0.04 |  | 0.578±0.04 | 0.576±0.04 |  | -1.51±0.8 | 0.27±0.6 |  | 1.79±1.0 | *n.s.* | |
| WM |  | aD | 1.043±0.05 | 1.037±0.04 |  | 1.041±0.04 | 1.035±0.04 |  | -0.23±0.4 | -0.23±0.3 |  | 0.05±0.5 | *n.s.* | |
|  | Contra- | FA | 0.377±0.03 | 0.377±0.03 |  | 0.379±0.03 | 0.372±0.03 |  | 0.59±0.4 | -0.84±0.4 |  | **-1.48±0.6** | **0.03** | |
|  |  | rD | 0.586±0.05 | 0.579±0.05 |  | 0.578±0.04 | 0.583±0.04 |  | -1.51±0.8 | 0.42±0.6 |  | 2.07±1.0 | *n.s.* | |
|  |  | aD | 1.043±0.05 | 1.037±0.04 |  | 1.041±0.04 | 1.036±0.05 |  | -0.23±0.4 | -0.09±0.3 |  | 0.22±0.5 | *n.s.* | |
| Superior | Ipsi- | FA | 0.360±0.02 | 0.365±0.03 |  | 0.359±0.02 | 0.361±0.02 |  | -0.35±0.4 | -0.91±0.3 |  | -0.78±0.6 | *n.s.* | |
| Temporal |  | rD | 0.600±0.05 | 0.585±0.04 |  | 0.592±0.03 | 0.592±0.04 |  | -1.30±1.0 | 0.99±0.5 |  | **2.41±0.9** | **0.02** | |
| WM |  | aD | 1.032±0.04 | 1.021±0.04 |  | 1.023±0.04 | 1.026±0.04 |  | -0.86±0.4 | 0.33±0.3 |  | **1.22±0.5** | **0.03** | |
|  | Contra- | FA | 0.360±0.02 | 0.368±0.02 |  | 0.359±0.02 | 0.364±0.03 |  | -0.35±0.4 | -0.99±0.3 |  | -0.70±0.6 | *n.s.* | |
|  |  | rD | 0.600±0.05 | 0.581±0.05 |  | 0.592±0.03 | 0.584±0.04 |  | -1.30±1.0 | 0.09±0.7 |  | 1.47±1.2 | *n.s.* | |
|  |  | aD | 1.032±0.04 | 1.019±0.05 |  | 1.023±0.04 | 1.017±0.04 |  | -0.86±0.4 | -0.30±0.4 |  | 0.62±0.6 | *n.s.* | |

**Bold:** FDR corrected *p* < 0.05

*n.s. =* not significant.

**S1 Appendix**

## The Parkinson’s Progression Markers Initiative Authors List:

#### **PPMI Steering Committee:** Kenneth Marek, MD^1^ (Principal Investigator); Danna Jennings, MD^1^ (Olfactory  Core, PI; Site Investigator); Shirley Lasch, MBA^1^; Caroline Tanner, MD, PhD^9^ (Site Investigator);  Tanya Simuni, MD^3^ (Site Investigator); Christopher Coffey, PhD^4^ (Statistics Core, PI); Karl Kieburtz, MD, MPH^5^ (Clinical Core, PI); Renee Wilson^5^; Werner Poewe, MD^7^ (Site Investigator); Brit Mollenhauer, MD^8^ (Bioanalytics Core, co-PI; Site Investigator); Douglas Galasko, MD^27^ (Bioanalytics Core, co-PI; Site Investigator); Tatiana Foroud, PhD^15^ (Genetics Coordination Core and Biorepository, PI); Todd Sherer, PhD^6^; Sohini Chowdhury^6^; Mark Frasier, PhD^6^; Catherine Kopil, PhD^6^; Vanessa Arnedo^6^

#### **PPMI Study Cores:** *Clinical Coordination Core*: Alice Rudolph, PhD^5^; Cynthia Casaceli, MBA^5^. *Imaging Core*: John Seibyl, MD^1^ (Principal Investigator); Susan Mendick, MPH^1^; Norbert Schuff, PhD^9^. *Statistics Core*: Chelsea Caspell^4^; Liz Uribe^4^; Eric Foster ^4^; Katherine Gloer PhD^4^; Jon Yankey MS^4^. *Bioinformatics Core*: Arthur Toga, PhD^10^ (Principal Investigator); Karen Crawford^10^. *Biorepository*: Paola Casalin^11^; Giulia Malferrari^11^. *Genetics Core*: Andrew Singleton, PhD^13^ (Principal Investigator). *Neuropsychological and Cognitive Assessments*: Keith A.  Hawkins, PsyD^14^

#### **PPMI Investigators:** David Russell, MD, PhD^1^; Stewart Factor, DO^16^; Penelope Hogarth, MD^17^; David Standaert, MD, PhD^18^; Robert Hauser, MD, MBA^19^; Joseph Jankovic, MD^20^; Matthew Stern, MD^12^; Lama Chahine, MD^12^; James Leverenz, MD^21^; Samuel Frank, MD^22^; Irene Richard, MD^23^;  Klaus Seppi, MD^7^; Holly Shill, MD^24^; Hubert Fernandez, MD^25^; Daniela Berg, MD^26^; Isabel Wurster MD^26^; Zoltan Mari, MD^28^; David Brooks, MD^29^; Nicola Pavese, MD^29^; Paolo Barone, MD, PhD^30^; Stuart Isaacson, MD^31^; Alberto Espay, MD, MSc^32^; Dominic Rowe, MD, PhD^33^; Melanie Brandabur MD^2^; James Tetrud MD^2^; Grace Liang MD^2^; Alex Iranzo, MD^34^; Eduardo Tolosa MD^34^; Shu-Ching Hu, MD, PhD^21^; Gretchen Todd^21^.

**PPMI Coordinators:** Laura Leary^1^; Cheryl Riordan^1^;  Linda Rees, MPH^2^; Alicia Portillo^17^; Art Lenahan^17^; Karen Williams^3^; Stephanie Guthrie, MSN^18^; Ashlee Rawlins^18^; Sherry Harlan^19^; Christine Hunter, RN^20^; Baochan Tran^12^; Abigail Darin^12^; Carly Linder^12^; Marne Baca^21^; Heli Venkov^21^; Cathi-Ann Thomas, RN, MS^22^; Raymond James, RN^22^; Cheryl Deeley, MSN^23^; Courtney Bishop BS^23^; Fabienne Sprenger, MD^7^; Diana Willeke^8^; Sanja Obradov^24^; Jennifer Mule^25^; Nancy Monahan^25^; Katharina Gauss^26^; Deborah Fontaine, BSN, MS^27^; Christina Gigliotti^27^; Arita McCoy^28^; Becky Dunlop^28^; Bina Shah, BSc^29^; Susan Ainscough^30^; Angela James^31^; Rebecca Silverstein^31^; Kristy Espay^32^; Madelaine Ranola^33^

#### **SAB (Industry Scientific Advisory Board):** Thomas Comery, PhD^35^; Jesse Cedarbaum, MD^36^; Bernard Ravina, MD, MSCE^36^; Igor D. Grachev, MD, PhD^37^; Jordan S. Dubow, MD^38^; Michael Ahlijanian, PhD^39^; Holly Soares, PhD^39^; Suzanne Ostrowizki, MD, PhD^40^; Paulo Fontoura, MD, PhD^40^; Alison Chalker, PhD^41^; David L. Hewitt, MD^41^; Marcel van der Brug, PhD^42^; Alastair D. Reith, PhD^43^; Peggy Taylor, ScD^44^; Jan Egebjerg, PhD^45^; Mark Minton, MD^46^; Andrew Siderowf, MD, MSCE^46^; Pierandrea Muglia, PhD^47^; Robert Umek, PhD^48^; Ana Catafau, MD,PhD^48^; Vera Kiyasova, MD, PhD^50^; Barbara Saba^50^

**PPMI Group Affiliation:**

^1^ Institute for Neurodegenerative Disorders, New Haven, CT.

^2^ The Parkinson’s Institute, Sunnyvale, CA.

^3^ Northwestern University, Chicago, IL.

^4^ University of Iowa, Iowa City, IA.

^5^ Clinical Trials Coordination Center, University of Rochester, Rochester, NY.

^6^ The Michael J. Fox Foundation for Parkinson’s Research, New York, NY.

^7^ Innsbruck Medical University, Innsbruck, Austria.

^8^ Paracelsus-Elena Klinik, Kassel, Germany.

^9^ University of California, San Francisco, CA.

^10^ Laboratory of Neuroimaging (LONI), University of Southern California.

^11^ BioRep, Milan, Italy.

^12^ University of Pennsylvania, Philadelphia, PA.

^13^ National Institute on Aging, NIH, Bethesda, MD.

^14^ Yale University, New Haven, CT.

^15^ Indiana University, Indianapolis, IN.

^16^ Emory University of Medicine, Atlanta, GA.

^17^ Oregon Health and Science University, Portland, OR.

^18^ University of Alabama at Birmingham, Birmingham, AL.

^19^ University of South Florida, Tampa, FL.

^20^ Baylor College of Medicine, Houston, TX.

^21^ University of Washington, Seattle, WA.

^22^ Boston University, Boston, MA.

^23^ University of Rochester, Rochester, NY.

^24^ Banner Research Institute, Sun City, AZ.

^25^ Cleveland Clinic, Cleveland, OH.

^26^ University of Tuebingen, Tuebingen, Germany.

^27^ University of California, San Diego, CA.

^28^ Johns Hopkins University, Baltimore, MD.

^29^ Imperial College of London, London, UK.

^30^ University of Salerno, Salerno, Italy.

^31^ Parkinson’s Disease and Movement Disorders Center, Boca Raton, FL.

^32^ University of Cincinnati, Cincinnati, OH.

^33^ Macquarie University, Sydney Australia.

^34^ Hospital Clinic of Barcelona, Barcelona, Spain.

^35^ Pfizer, Inc., Groton, CT.

^36^ Biogen Idec, Cambridge, MA.

^37^ GE Healthcare, Princeton, NJ.

^38^ AbbVie, Abbot Park, IL.

^39^ Bristol-Myers Squibb Company.

^40^ F.Hoffmann La-Roche, Basel, Switzerland.

^41^ Merck & Co., North Wales, PA.

^42^ Genentech, Inc., South San Francisco, CA.

^43^ GlaxoSmithKline, Stevenage, United Kingdom.

^44^ Covance, Dedham, MA.

^45^ H. Lundbeck A/S.

^46^ Avid Radiopharmaceuticals, Philadelphia , PA.

^47^ UCB Pharma S.A., Brussels, Belgium.

^48^ Meso Scale Discovery.

^49^ Piramal Life Sciences, Berlin, Germany.

^50^ Servier.
